# Vaccinia Virus Vector Bivalent Norovirus Vaccine

**DOI:** 10.3390/v17020237

**Published:** 2025-02-09

**Authors:** Yunbo Bai, Xi Wu, Yanru Shen, Liangliang Wang, Ziqi Cheng, Yeqing Sun, Hao Wu, Qingfeng Zhang, Ziqi Sun, Chenchen He, Binfan Liao, Weijin Huang, Huanzhang Xia

**Affiliations:** 1 School of Life Science and Biopharmaceutics, Shenyang Pharmaceutical University, Shenyang 110016, China; baiyunbo@nidfc.org.cn (Y.B.);; 2 State Key Laboratory of Drug Regulatory Science, Institute for Biological Product Control, National Institutes for Food and Drug Control (NIFDC), Division of HIV/AIDS and Sexually Transmitted Virus Vaccines, Beijing 102629, China

**Keywords:** norovirus, poxvirus vector, bivalent vaccine, immune response

## Abstract

Norovirus is a major etiological agent of nonbacterial gastroenteritis around the world. Due to its in vitro culture complexity, high genome diversity, and the lack of cross-reactive immunity between genogroups, there is an unmet urgent need for polyvalent norovirus vaccines that provide broad-spectrum protection, and no vaccine has gained global approval to date. In this study, we constructed a bivalent norovirus vaccine, based on the highly attenuated poxvirus [strain VG9] vector, expressing the major capsid protein VP1 from genotypes GII.4 and GII.17. VG9-NOR exhibited a comparable replication ability to the authentic virus while preserving good safety. After the intramuscular and intranasal immunization of mice, VG9-NOR induced high IgG- and IgA-binding antibody (Ab) titers against GII.4 and GII.17, increased the secretion of GII.4 and GII.17-specific HGBA-blocking antibodies, and enhanced GII.17-specific mucosal immunity. Furthermore, VG9-NOR also induced a Th1-mediated cellular response. These results demonstrate that the polyvalent poxvirus vector vaccine expressing VP1 variants from different subtypes is able to elicit effective protection. Our study highlights the VG9 vector as a highly promising candidate for the development of polyvalent norovirus vaccines.

## 1. Introduction

Norovirus, also known as Norwalk virus, was first reported in 1972 as the cause of an outbreak of acute gastroenteritis in the United States [1]. Hosts can be infected via the fecal–oral route or contact with aerosols from vomiting patients [2,3]. The high infectivity and effective transmission have maintained the worldwide norovirus pandemic to date. There are currently 10 genogroups of noroviruses [GI-GX], five of which infect humans [GI, GII, GIV, GVII, and GIX] [4]. Notably, GII.4 is the most widespread genotype that accounts for 70–80% of all norovirus cases, and is currently the dominant strain associated with numerous recent NoV outbreaks worldwide [5,6,7,8,9,10]. However, no cross-reactive immunity was detected between GII.4 and GII.17 [11,12], emphasizing the essential need to develop bivalent vaccines against these genotypes.

Norovirus belongs to the calicivirus family, possessing a 7.5 kb uncoated positive-sense RNA genome encoding three open reading frames [ORF1-3]. ORF1 encodes a polyprotein, which is cleaved into non-structural proteins after translation. ORF2 is responsible for the expression of the major capsid protein VP1, which consists of a shell [S] domain and a protruding [P] domain. ORF3 encodes the minor capsid protein VP2 [13]. Notably, VP1 alone can self-assemble into virus-like particles [VLPs] [14], which have become the focus of norovirus vaccine development. Norovirus VLPs have been successfully produced in various systems, including *E. coli* [15], *Pichia pastoris* [16], insect cells [17], and mammalian cells [293T] [18], as well as vesicular stomatitis virus [VSV] [19] and adenovirus vectors [20]. Currently, several norovirus vaccines are being tested in clinical trials, including a quadrivalent recombinant norovirus vaccine [NCT04563533] [21], adenovirus-based VXA-NVV-201,202 norovirus vaccines [NCT05212168, NCT05626803] [22], recombinant norovirus bivalent vaccines [National Vaccine & Serum Institute, NCT04941261, NCT05916326] [23], bivalent vaccines against genotypes GI.4/GII.4 [NCT05508178] [24], a recombinant hexavalent vaccine [NCT05805618] [25], an mRNA-1403/mRNA-1405 polyvalent vaccine [NCT05992935] [26], and recombinant tetravalent vaccines [CXSL2300464 CXSL2300465] [27]. However, testing of the bivalent vaccine developed by Takeda Pharmaceutical has been terminated due to an insufficient therapeutic effect. In addition, a trivalent vaccine against the rotavirus capsid protein VP6 carries the norovirus GII.4 VP1 [28] and a *Lactobacillus*-based vaccine against VP1 [29] is being developed.

The vaccinia virus vector has a large gene-carrying capacity, with a maximal insertion length of 25 kb [30], and has been applied in HIV vaccine development [31]. However, the efficiency of the vaccinia virus vector for the norovirus vaccine has yet to be determined. In this study, we constructed a bivalent norovirus vaccine using the VG9-attenuated strain of vaccinia virus as the vector, named VG9-NOR. The VG9 strain was obtained via three cycles of plaque purification of the Tian Tan strain of the vaccinia virus [VVT], which has the advantages of low toxicity, high intake capacity, and immunogenicity, while providing a sustained high expression of the introduced genes [32]. The VP1 coding sequences of the GII.4 and GII.17 strains were placed under the control of the ELO160 and PE/L promoters. In this study, we assessed the VP1 gene expression level, genetic stability, safety of VG9-NOR, and immunogenicity of the VG9-NOR bivalent vaccine.

## 2. Materials and Methods

### 2.1. Cells

Human embryonic kidney [HEK] 293T cells, baby hamster kidney [BHK] 21 cells, and Vero cells were cultured in Dulbecco‘s modified Eagle medium [DMEM] supplemented with 10% of fetal bovine serum [Gibco-Life Technologies, Carlsbad, CA, USA], at 37 °C in a humified incubator with 5% CO_2_. All cells originated from the Division of HIV/AIDS and Sexually Transmitted Virus Vaccines, National Institutes for Food and Drug Control, China.

### 2.2. Plasmid Construction

To construct the shuttle vector, the VP1 coding sequences of GII.4 [GenBank: AID60887.1, Hu/GII.4/Beijing/PKUPH-07-02/inpatient/2013/CHN] and GII.17 [GenBank: YP_009518836.1, Norovirus Hu/GII.17/HKG/2014/CUHK-NS-456] were codon-optimized and inserted into plasmids with the inducible promoters ELO160 [33] and PE/L [34]. The plasmid also contained a P11-induced late-expression enhanced green fluorescent protein (EGFP) as a screenable marker, both ends of EGFP containing a LoxP sequence [35]. To ensure the plasmid was inserted between the TJ2R and TJR3 sites of the VG9 strain, both ends of the constructed fragments carried an 819 bp TJR homologous sequence in the PE/O-NOR [GENERAL BIOL synthetic] and Cre [Cyclization Recombination Enzyme] plasmids (GENERAL BIOL synthetic).

### 2.3. VG9 Virus Strain and Construction of the Norovirus Vaccine

The VG9 virus strain (stored at the NIFDC) was obtained via 3 rounds of plaque purification from VVT, exhibiting reduced toxicity while maintaining good immunogenicity [32].

The 293T cells were seeded at a density of 2 × 10^6^ and infected with 0.01 MOI VG9 for 1–2 h. After infection, the cells were transfected with 10 μg of PE/O-NOR plasmid using Lipofectamine 3000 [Invitrogen, Carlsbad, CA, USA] and incubated for 48 h [36]. The cells were then repeatedly freeze–thawed to release the virus. Vero cells were used to select the recombinant virus via 10 rounds of plaque purification until a monoclonal fluorescent plaque was observed. The obtained recombinant virus was named VG9-NOR-EGFP.

Vero cells were transfected with the CRE-expression plasmid using Lipofectamine 3000 and then infected with VG9-NOR-EGFP. Following 72 h post-infection, non-EGFP expressing cells were harvested and purified 6 times to obtain VG9-NOR.

### 2.4. VP1 Expression on VG9-NOR

BHK21 cells were infected with 5 MOI of VG9-NOR or VG9 [negative control] and incubated for 48 h. Then, the cells were collected by scratching from the plates and centrifuged at 13,000× *g* for 5 min, after which 100 µL of lysis buffer [containing 1 mM PMSF] was added to the cell debris to release intracellular proteins. After 10 min of incubation on ice, protein samples were collected by refrigerated centrifugation [13,000× *g*, 10 min] and separated by SDS-PAGE. Western blot analysis was performed using primary rabbit polyclonal anti-GII.4 and GII.17 VP1 antibodies [1:2000 dilution, Beijing Health Guard Biotechnology, Beijing, China], followed by a secondary horseradish peroxidase [HRP]-conjugate anti-rabbit antibody [1: 10,000 dilution, CWBIO: CW0103S], and visualized using the enhanced Horseradish Peroxidase [HRP]-luminol chemiluminescent system [ECL Plus, Thermo scientific, Rockford, IL, USA].

### 2.5. Sequence Validation of the Recombinant Virus

Viral DNA extraction was performed using an DNA/RNA extraction kit [Transgene, D2410-50T] according to the manufacturer’s instructions. Primers were designed to amplify the VP1 gene of GII.4 [forward: 5′-TTTTATTTTTTTTTTTTGGAATATAAATATCC-3′, reverse: 5′-ACAGGGCTCTCCTCCT-3′] and GII.17 [forward: 5′-CTGGGCTCTTCTTCTGC-3′, reverse: 5′-AAAAATTGAAATTTTATTTTTTTTTTTTGGAATATAAAT-3′]. DNA samples were analyzed by 1% agarose gel electrophoresis.

### 2.6. Genetic Stability of VG9-NOR

After the isolation of a single clone, the third generation [P3] of VG9-NOR was collected and prepared in the stock solution for the genetic stability assay. Firstly, BHK-21 cells were infected with 0.05 MOI of VG9-NOR. Following 72 h post-infection, the cells were scratched from plates and centrifuged at 13,000× *g* for 10 min. The supernatant was collected and used for subsequent infection with 0.05 MOI of VG9-NOR for a total of 6 passages. Western blot analysis was performed in each passage to assess VP1 expression. Protein stability and changes were evaluated using primary anti-GII.4 and GII.17 VP1 antibodies, combined with VG9-E3 [VG9 immunized serum, 1:5000] and GAPDH [Glyceraldehyde-3-phosphate dehydrogenase] [Proteintech HRP-81640-5] as positive and negative control, respectively.

### 2.7. Growth Curve

The Vero, BHK-21, and HeLa cells were infected with 0.05 MOI of VG9-NOR and VG9 for 2 h, after which they were washed with DMEM 3 times and resuspended in DMEM with 2% FBS. After 12, 24, 48, and 72 h of incubation, scrapped cells were frozen and lysed [36]. Virus titrations were performed as previously described [37].

### 2.8. Chick Embryo Chorioallantoic Membrane [CAM] Model

The VG9 and VG9-NOR viral suspensions were adjusted to 1 × 10^6^ PFU/mL and respective 100 μL was collected to inoculate the CAM of specific-pathogen-free (SPF) chick embryos. The size, structure, and number of the pox were compared 3 days post-inoculation.

### 2.9. Skin Pathogenicity Assay in Rabbits

The VG9-NOR and VG9 strains were subjected to 10-fold gradient dilution 5 times and intracutaneously injected into female New Zealand white rabbits (average size approximately 2.5 kg) on both sides of the spine. VG9-NOR was injected on the left side while VG9 was administered on the right. Rabbit received intracutaneous injections of both the VG9-NOR and VG9 strains, each strain injected at 5 sites, each dilution injection comprising 0.1 mL. The diameters of red pocks and necrotic lesions were measured 3 days post-infection and monitored for consecutive 9 days.

### 2.10. Vector Safety Evaluation

Mice were intramuscularly and intranasally injected with 2 × 10^7^ PFU VG9-NOR and VG9 strains. Following 14 days post-injection, the hearts, livers, spleens, lungs, kidneys, and brains were collected for histological analysis. Tissues were embedded in paraffin and sliced into 3 µm-thin sections, followed by H&E staining.

### 2.11. Mice

The animal ethics committee of the National Institute for Food and Drug Control (NIFDC), Beijing, China, approved the studies on immunogenicity under permits No. NIFDC (F) 2024(B)005 and NIFDC (F) 2024(B)050. The animal immunization procedures adhered to international guidelines and Chinese law.

Female BALB/c mice aged 6–8 weeks were divided into two groups which were intranasally or intramuscularly administered a high [1 × 10^7^ PFU/mL, 100 µL] or low dose [1 × 10^6^ PFU/µL, 100 µL], respectively. The VG9 strain was used as the negative control. Mice received a booster dose 14 days after the first immunization. Following 14 days after the second immunization, the mice were sacrificed through CO_2_ inhalation. For intracellular cytokine staining [ICS], the mouse spleen was removed. Weight was measured one day before and thirteen days after the first dose of vaccine, while the blood and feces were collected fourteen days after each immunization for humoral immunity evaluation.

### 2.12. ELISA

The 96-well plates [Corning:3590] were coated with 1 μg/mL GII.4 and GII.17 VLP, 100 µL per well, and incubated at 4 °C overnight. The coated plates were then blocked with 5% skimmed milk. After 2 h, the plates were washed with PBST [containing 0.05% Tween 20], after which the mouse serum was added to the plate and incubated for 1 h at 37 °C. Subsequently, the plates were washed 3 times, after which the HRP-labelled goat-anti-mice IgG [Boster: BA1050]/Goat Anti-Mouse IgA alpha chain (HRP) [Abcam: ab97235] was added and incubated for 1 h, and then washed 5 times. Following the addition of 100 µL TMB [3,3′,5,5′-Tetramethylbenzidine] substrate I [Biomart:TMB-S-001], the plate was incubated at 37 °C for 25 min, after which the reaction was terminated by adding the stop buffer [Solarbio:C1059] and the absorbance at 450 nm (A_450_) was measured. The binding antibody titer was defined as the reciprocal of the highest serum dilution [absorbance ≥ 2.1 OD_450_ units higher than the control].

### 2.13. Antigen-Blocking Antibody [HBGA] Binding Assay

High-binding 96-well plates were coated with 5 μg/mL PMG [mucin from porcine stomach, Type III] overnight at 4 °C. After washing with PBST [containing 0.05% Tween 20] 3 times, the plates were blocked with 2% Bovine Serum Albumin [BSA] for 2 hrs, and then washed with PBST 3 times. At the same time, mouse serum was mixed with GII.4 or GII.17 VLP and incubated at 25 °C for 1 h. The mixture was then added to the blocked plates, and incubated for 1 h followed by washing with PBST 3 times. The HRP-labelled mouse anti-norovirus antibody [Health Guard Biotechnology] was added to the plate and incubated for 1 h, after which the plates were washed 5 times using PBST. Following 10 min after adding the TMB substrate, the A_450_ was recorded on a SpectraMax M5 plate reader. Maximum binding was determined using the VLP without mouse serum. The 50% blocking antibody titer [BT50] was defined as the reciprocal of the highest serum dilution that blocks 50% of the maximum VLP binding.

### 2.14. Norovirus-Specific Cellular Immune Response

The extracted mouse spleens were placed on a Petri dish containing 1640 medium [Gibco 6187003] with 10% FBS [Fetal Bovine Serum]. To separate mouse spleen lymphocytes, the spleen tissue was macerated and passed through a 70 μm-gap-size cell strainer. Red blood cells were removed by adding ACK lysis buffer [Solarbio:R1010] for 3–5 min. The separated mononuclear cells were adjusted to a density of 2 × 10^6^, added to 96-well plates, and mixed with 30 µg/mL VLP of GII.4 or GII.17 [Health Guard Biotechnology, Beijing, China]. After 2 h of stimulation, GolgiPlug Protein Transport Inhibitor [BD] was added to each well and incubated overnight. The next day, lymphocytes were collected by centrifugation at 900× *g* for 5 min. The cells were then stained with Zombie reagent [BioLegend, 423106] at room temperature for 15 min. Cytokine surface staining was conducted using 1 μL of FITC anti-mouse CD3 [BioLegend, 1002404], 1 μL of PercP/Cyanine5.5 anti-mouse CD4 [BioLegend, 116012], and 1 μL of Brilliant Violet 510 anti-mouse CD8a [BioLegend, 100752], followed by fixation [Fixation: BD, 554722]. Finally, the intracellular cytokines IFN-γ [BioLegend, 505826], IL-2 [BioLegend, 503808], IL-4 [BioLegend, 504106], and TNF-α [BioLegend, 506328] were stained at room temperature. Then, cells were centrifuged at 900× *g* for 5 min. Spleen cell debris was resuspended in 300 μL of PBS.

### 2.15. Mouse Fecal IgA Antibodies

The fecal extraction solution was prepared by adding 0.1% Tween 20 and protease inhibitor [Yeasen, 20123ES10] into sterile PBS. The collected fecal samples were weighted, suspended in extraction solution at ratio of 1: 10 [*w*/*v*], and incubated at 4 °C for 30 min, followed by 1 min of vortexing for throughout mixing and another 30 min of incubation at 4 °C. The samples were centrifugated at 13,000× *g* for 10 min, after which the supernatants were collected and subjected to another centrifugation at 13,000× *g* for 10 min. The resulting supernatants were stored at −20 °C for future use.

The fecal IgA Abs were evaluated via ELISA. After coating with GII.4 and GII.17 VLP overnight, the 96-well plates were filled with 20-fold diluted extracted fecal samples. After 20 min of incubation, Goat Anti-Mouse IgA alpha chain (HRP) [Abcam: ab97235] was added to each well, and incubated for 1 h. The A_450_ readings were recorded after adding 100 µL of TMB substrate I. The baseline titer was calculated based on the mean A_450_ value of five unimmunized mice +3 standard deviations. The A_450_ of immunized mice ≥ baseline titer value was considered positive, and vice versa.

### 2.16. Statistical Analysis

All data analyses were carried out using GraphPad Prism 10 software. Statistical analysis was performed using ANOVA and Tukey’s test.

## 3. Results

### 3.1. Construction of VP1-Expressing Recombinant VG9-NOR

**Figure 1:**
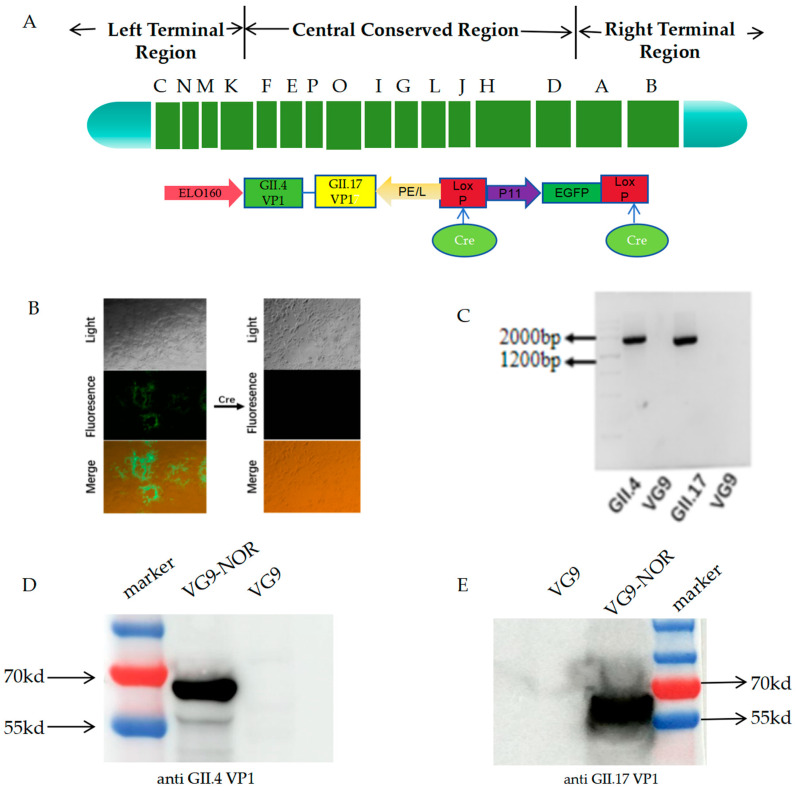
Construction and validation of vaccinia vector bivalent norovirus vaccine: (**A**) The gene segment that was inserted into VG9 and shuttle plasmid construction. Different domains were labelled with capital letters. Terminal regions were located on both ends of the gene segment. The VP1 protein sequences of the GII.4 and GII.17 strains were expressed using the ELO160 and PE/L promoters, respectively. The sequences of the VG9 TK site [J2R] were used as the homologous arm. An EGFP expression cassette driven by the P11 promoter was inserted between the CRE-recognition sites d. (**B**) VG9-NOR-infected Vero cells before and after EGFP knockout, observed using an inverted fluorescence microscope. (**C**) Validation of VP1 gene insertion in VG9-NOR by PCR. (**D**,**E**) Evaluation ofVP1 protein expression by Western blot analysis. BHK21 cells were infected with 5 MOI of VG9-NOR and VG9 for 24 h and then lysed. Protein samples were separated using 10% polyacrylamide SDS-PAGE and the specific bands detected using rabbit polyclonal antibodies against GII.4 and GII.17 VP1, respectively.

To construct the GII.4 and GII.17 bivalent norovirus vaccine, the VG9 strain was used as the vector to express the VP1 protein of both genotypes. The structure of the constructed bivalent VG9-NOR is shown in Figure 1A. The early and late promoter ELO160 plays a role in immune regulation [33] and was introduced to induce the expression of GII.4 VP1, while the expression of GII.17 VP1 was driven by the promoter PE/L [34]. To prevent expression interference, the two promoters were arranged in a “head-to-head” manner. The screenable marker EGFP was inserted between two loxP sites and its expression was driven by the p11 promoter [38]. This segment was then inserted between the J2R and TJ3R sites in the genome of the vaccinia virus. The recombinant virus VG9-NOR-EGFP was obtained through multiple consecutive rounds of plaque purification. Subsequently, the VG9-NOR-EGFP infected cells were transfected with the plasmid expressing Cre recombinase (Figure 1B), after which six additional rounds of purification were performed to obtain the EGFP knockout recombinant VG9-NOR. The insertion was validated by PCR (Figure 1C). A Western blot analysis confirmed that GII.4 and GII.17 VP1 proteins were both successfully expressed in VG9-NOR (Figure 1D,E).

### 3.2. Stability of the VG9-NOR Bivalent Vaccine

**Figure 2:**
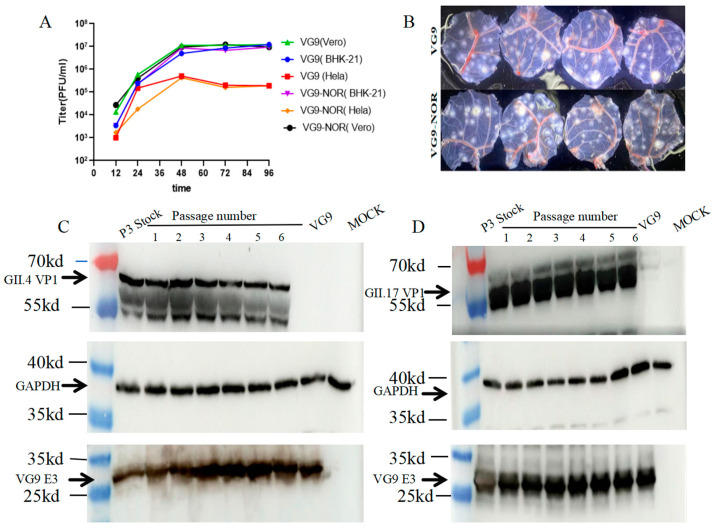
Genetic stability of VG9-NOR: (**A**) Growth curve of VG9-NOR and VG9 in different cells. Vero, BHK-21, and HeLa cells were infected with 0.05 MOI of VG9-NOR. Viral titers were determined using the plaque assay at 12 h, 24 h, 36 h, 48 h, and 72 h post-infection (the third generation to establish the growth curve). (**B**) Chick embryo chorioallantoic membrane (CAM) assay. The CAM of chick embryos was inoculated with 1 × 10^6^ PFU of VG9 or VG9-NOR. The upper images show the VG9-induced pocks and the lower images show the VG9-NOR-induced pocks (n = 4). (**C**,**D**) Western blot analysis of VP1 expression in different generations. BHK-21 cells were infected with 0.05 MOI of P3 generation VG9-VOR. Following cell lysis, the virus was passaged for 6 generations. The protein samples of each generation were separated by 10% acrylamide SDS-PAGE and detected using rabbit polyclonal antibodies against GII.4 and GII.17 VP1, combined with mouse polyclonal antibodies against VG9-E3 and GAPDH. The E3 protein was used as loading control. Black arrows indicate the location of GII.4 and GII.17 VP1, GAPDH, and VG9 E3. The approximate molecular weight is indicated on the left.

To ensure that the insertion of the VP1 coding sequence did not adversely affect viral replication and genetic stability, we infected Vero, BHK-21, and HeLa cells with 0.05 MOI of VG9-NOR. The recombinant virus showed a comparable replication ability to the VG9 strain (Figure 2A). We then compared the morphology of pox on the chorioallantoic membrane (CAM) of chick embryos induced by VG9 and VG9-NOR, finding no noticeable difference in size, shape, or number (Figure 2B). These results indicate that the replication capability was preserved in the VG9-NOR strain. Since gene loss frequently occurs during the passaging of the vaccinia virus due to gene deletion, frameshift, or nonsense mutations [39], we investigated the genetic stability of the inserted sequence. The third-generation virus after purification was obtained and used to infect BHK-21 cells. The cell supernatant was collected and passaged for six consecutive generations. Protein expression was evaluated by a Western blot analysis. The results showed that the VP1 proteins of the GII.4 and GII.17 strains were stably expressed in all generations, which confirmed the genetic stability of VG9-NOR.

### 3.3. Safety Evaluation of VG9-NOR Bivalent Norovirus Vaccine

**Figure 3:**
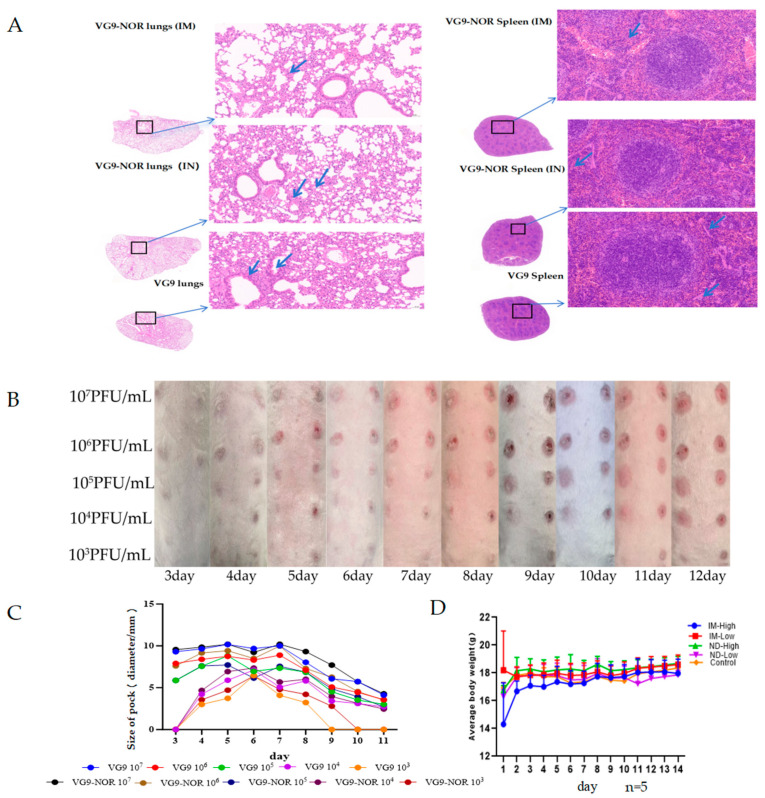
Safety evaluation of VG9-NOR bivalent norovirus vaccine: (**A**) Pathological analysis of the lungs and spleen of mice following intramuscular vaccination. (**B**) Skin condition monitoring following the administration of different doses of VG9/VG9-NOR in rabbits. Concentration from top to below: 10^7^, 10^6^, 10^5^, 10^4^, and 10^3^ PFU. VG9-NOR was injected at the left side of the spinal cord, and the right side was injected with VG9. (**C**) Red pock lesion size monitoring in the injected rabbits. (**D**) Weight changes after VG9-NOR injection [days 2–13], where day 1 represents the pre-infection weight. Control-group mice received 100 μL of VG9 suspension, intranasally or intramuscularly in high dose [1 × 10^7^ PFU] or low dose [1 × 10^6^ PFU].

The TTV-derived VG9 strain has low toxicity and causes fewer side effects [32]. To evaluate the safety of VG9 and VG9-NOR, we conducted pathological sectioning, rabbit skin pockmark test, and body weight analysis in mice after vaccination. Firstly, 14 days after a single dose of the vaccine, we performed pathological sectioning on the heart, liver, spleen, lung, kidney, and brain of mice. Figure 3A shows the pathological results of the lung and spleen following intramuscular vaccination with VG9-NOR, intranasal VG9-NOR, and VG9 (see Supplementary Materials). There were no obvious lesions in the lungs and spleen, with only slight lymphocyte infiltration. This suggests that the pathological changes caused by VG9 and VG9-NOR in mice are minimal. Secondly, we carried out the rabbit pockmark test, which revealed that there was no significant difference in the size of the pockmarks formed by the two viral strains, but the pockmarks induced by low-dose VG9-NOR receded faster in the last three days (Figure 3B,C). This may be due to a slightly weaker replication ability of VG9-NOR in rabbit skin caused by the insertion of exogenous genes. Lastly, we further analyzed the impact of VG9-NOR and VG9 on the body weight of mice (Figure 3D). The results showed that the changes in the body weight of mice were not significant, indicating that VG9 and VG9-NOR had a minimal impact on the general health of mice. In conclusion, VG9-NOR showed a similar safety profile to VG9 in rabbits and mice. These results suggest that VG9-NOR, as a vaccine candidate strain, has good safety and a low risk of side effects in the animal model tested.

### 3.4. VG9-NOR-Induced Robust Humoral and Cellular Immunity

**Figure 4:**
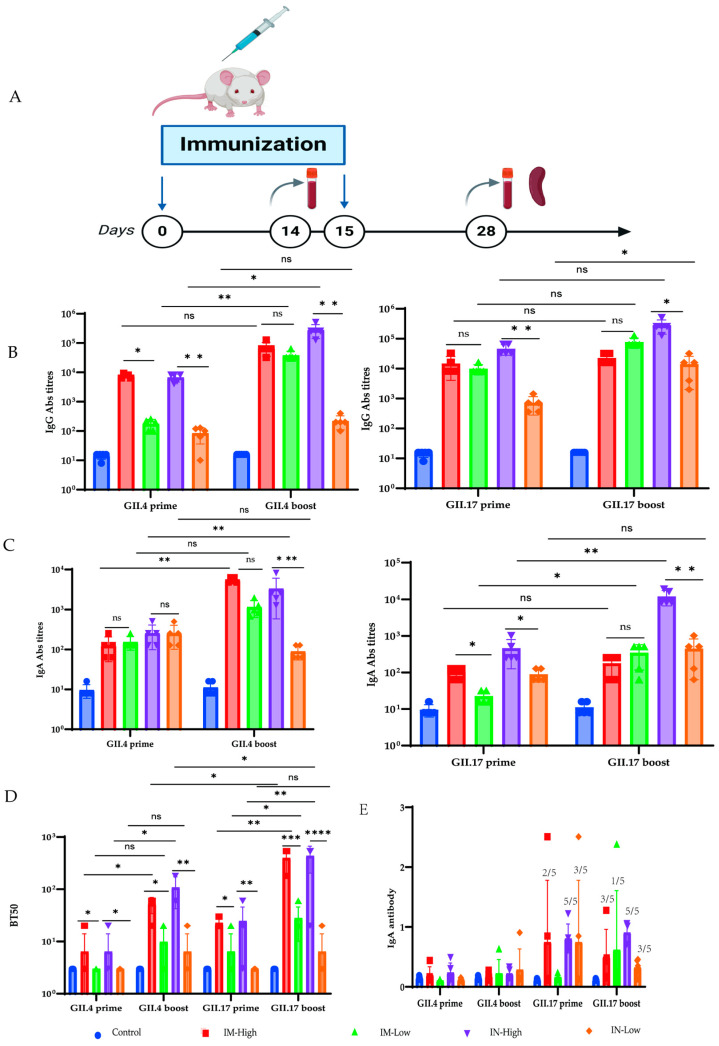
Induction of humoral immunity by VG9-NOR bivalent vaccine: (**A**) Mouse immunization procedure. (**B**) Serum IgG binding Ab titer induced by VG9-NOR immunization. (**C**) Serum IgA Ab titer induced by VG9-NOR immunization. (**D**) HGBA-blocking antibody production in response to the vaccine. The IgA- and IgG-binding Abs titers were determined according to the colorimetric absorbance (A_450_) value of the maximum antibody dilution in ELISA, which was 2.1-fold higher than the blank control. The reciprocal of this dilution was used as the binding antibody titer. BT50 values were calculated based on the reciprocal of the highest dilution of the A_450_ value just below the median A_450_ of the positive and negative control groups. The bars indicate means ± SEM. Each dot represents a single mouse. (**E**) Fecal IgA Abs in the VG9-NOR immunized mice. The number of fecal IgA-Abs-positive mice/total number of mice is labelled above each bar. The baseline titer value was calculated based on the mean A_450_ value of five unimmunized mice +3 standard deviations. The A_450_ of immunized mice ≥ baseline titer value was considered positive, and vice versa [ns, not significant; * *p* < 0.05, ** *p* < 0.005, *** *p* < 0.0001, and **** *p* < 0.0001; each dot represents a mouse, n = 5].

To assess the humoral response induced by VG9-NOR, BALB/c mice were intramuscularly or intranasally immunized with 1 × 10^6^ [high dose] or 1 × 10^5^ [low dose] PFU of VG9-NOR (Figure 4A), and boosted after 14 days. We conducted ELISA 14 days after each round of immunization to evaluate the IgG (Figure 4B) and IgA Abs (Figure 4C). The results showed that the high-dose intranasal immunization elicited the highest antibody titers against GII.4 and GII.17, respectively. In addition, HGBA blockage is also an essential indicator for assessing norovirus vaccines [40]. We thus performed an HGBA blockage assay and observed that VG9-NOR immunization was able to induce antibodies blocking HGBA, whereby the intranasal route showed the best performance. Intestinal IgA Ab is one of the major antibodies that can prevent norovirus infection. Accordingly, we examined mouse fecal IgA Abs 14 days after the first dose and the booster dose. The intranasally immunized mice showed a 100% positivity rate for fecal IgA Abs against GII.17 (Figure 4E). The mice immunized via the intramuscular route showed a rise in the fecal IgA Abs positivity rate from 40 to 60% after boosting. However, no GII.4-specific fecal IgA Ab was observed in any of the immunized mouse. These results indicate that intranasal administration of the high dose is a preferable and safe way for VG9-NOR immunization, which can induce high IgG, and IgA, as well as fecal IgA antibodies shortly after immunization. The difference between high and low doses of HGBA-blocking antibodies against GII.4 and GII.17 was statistically significant, with higher antibody Abs in the high-dose group. In addition, the IgG- and IgA-binding antibodies titers against GII.4 and GII.17 after the second dose were higher than after the initial dose, but there was no significant difference in IgG-binding Ab against GII.17 following the first and second dose.

**Figure 5:**
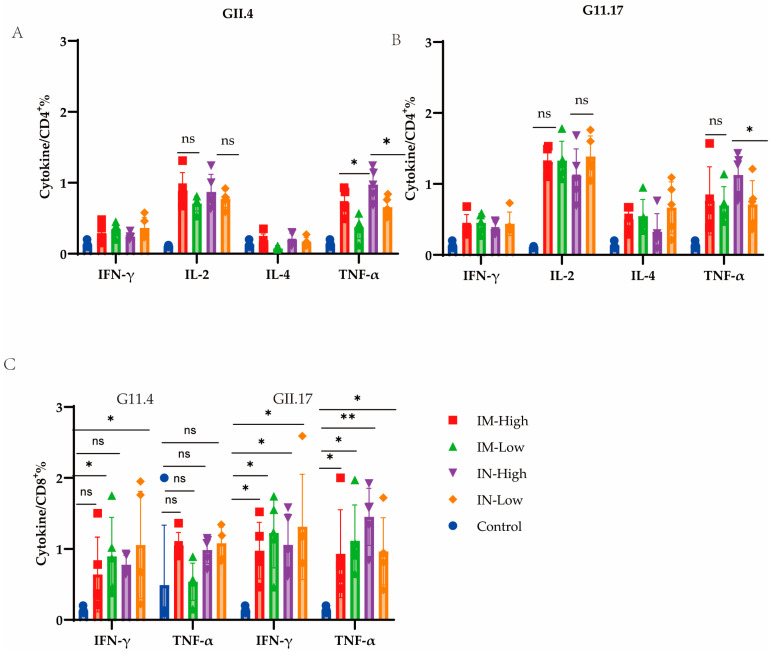
Cellular immunity induced by the VG9-NOR bivalent vaccine. (**A**) Percentage of IFN-γ-, IL-2-, IL-4-, and TNF-α-secreting CD4^+^ cells against GII.4 (**B**) and GII.17 (**C**). Percentage of IFN-γ-and TNF-α-secreting CD8+ cells against GII.4 and GII.17 [ns, not significant; * *p* < 0.05, ** *p* < 0.005; each dot represents a mouse, n = 5].

Cellular immunity is strongly activated after a norovirus infection to eliminate the virus [41]. We, therefore, evaluated if VG9-NOR induced a cellular immune response in mice. Mouse splenocytes were separated 14 days after the booster dose. We observed that VG9-NOR activated the IL-2- and TNF-α-secreting CD4^+^ T-cell response (Figure 5A,B). An analysis of the TNF-α and IFN-α secretion by CD8^+^ T cells (Figure 5C) revealed that there was no significant difference between the cells induced by GII.4 and the blank control, while there was a significant difference between the cells induced by GII.17 and blank control.

## 4. Discussion

Norovirus infection is one of the main causes of acute gastroenteritis around the world. The lack of cross-immunity between prevalent strains has led to an unmet, yet urgent, need for a polyvalent vaccine that provides wider protection. In this study, we selected the low-pathogenicity vaccinia strain VG9 as the vector, into which we inserted the VP1 genes of prevalent strains GII.4 and GII.17 VP1 to develop the bivalent norovirus vaccine VG9-NOR. GII.4 is a widespread norovirus genotype that is continuing to mutate [42,43], while GII.17 is currently the dominant strain associated with numerous recent NoV outbreaks worldwide [6,7,8,9,10].

Vaccinia vectors are widely used in the development of polyvalent vaccines, primarily because they can effectively induce mucosal immunity, and possess high genomic stability with a concomitant low host–genome integration risk, while allowing the insertion of large gene fragments. The recently marketed preventive vaccinia vector vaccine, a non-replicating Ankara strain, has exhibited high vaccine safety. The vaccinia vector vaccines in the clinical trials showed no adverse effects, including those against Zika virus [44], Ebola virus [36], and chikungunya fever virus [45]. Here, we evaluated the safety of VG9-NOR by comparing the mouse organ histology, skin pox lesions, and mouse weight changes following VG9-NOR immunization. Our results confirmed that VG9-NOR has good safety in both mice and rabbits.

The World Health Organization [WHO] has not provided evaluation guidance for norovirus vaccines. Thus, we assessed the immunogenicity of VG9-NOR by measuring various immune responses. The VG9-NOR bivalent vaccine was able to simultaneously induce systemic mucosal immunity against both GII.4 and GII.17, including high titers of IgA- and IgG-binding Abs, which are essential antibodies that defend against norovirus infection [46]. Compared with the norovirus vaccine developed by Jiang et al. [20], high-dose intranasal immunization with VG9-NOR elicited higher IgG [titer:2^20^] and IgA [titer:2^25^] Abs titers. The VG9-NOR-induced GII.4-specific IgA antibody was higher than that of the adenovirus vector vaccine, whereas intranasal administration induced a slightly lower IgG antibody count than the adenovirus vector vaccine [maximal titer 10^5^]. As shown in Figure 3C,D, our purpose was to study the stability of the antigen expression during passage. However, multiple bands were observed in GII.4, and the molecular weights of GII.4 and GII.17 were also inconsistent. For the multiple bands in GII.4, we proposed that it may be due to the hydrolysis of its N-terminal, as a similar phenomenon had also been observed by other researchers using VSV to express the VP1 protein of GII.4 [19]. With regard to the difference in molecular weight between GII.4 and GII.17, we speculated that this may be due to differences in the reactivity of polyclonal antibodies used against different antigens. Specifically, polyclonal antibodies of GII.4 may be more responsive to intact virus-like particles (VLPs), while polyclonal antibodies of GII.17 may be more active to N-terminal-degraded fragments. Meanwhile, we found that the GII.17 expression level was higher, which was consistent with our immunological results that the fecal IgA antibody and HGBA-blocking antibody in GII.17 were all higher than that in GII.4.

Since the histo-blood group antigen [HBGA] antibody is one of the key factors in assessing the protectivity of norovirus vaccines [47], we demonstrated that VG9-NOR can induce HBGA antibodies with a comparable BT50 value to the vaccines developed by Jiang and colleagues [20].

In addition to humoral responses, cellular immunity is also essential in response to norovirus infection [48]. We therefore investigated if VG9-NOR activated cellular immunity. It was found that VG9-NOR induced a Th1-biased response in mice. After stimulation with genotype-specific VLPs, there was a higher secretion of IL-2 and TNF-α compared to IFN-γ and IL-4, suggesting a strong CD4+ cellular response. Atochina-Vasserman et al. [49] developed a bivalent norovirus mRNA vaccine, which also induced double-positive CD4^ +^ T cells as well as polyfunctional CD8 ^+^ T cells, which was consistent with our results.

In this study, we evaluated the immunogenicity of the VG9-NOR vaccine, particularly against GII.4 and GII.17 norovirus. However, there are some limitations to our study, particularly in the evaluation of vaccine immunogenicity and safety. First, we did not use a non-human primate (NHP) model to evaluate the immunogenicity of the VG9-NOR vaccine, which is considered the most reliable platform for evaluating vaccine immunogenicity. This limitation may affect the accuracy of our assessment of the vaccine’s actual protective effect in humans. Secondly, we found that the VG9 strain induced pox lesions on the skin of rabbits. Therefore, before conducting human clinical studies, further safety tests in non-human primates are needed. Finally, we only explored intranasal and intramuscular immunization methods, while subcutaneous methods and oral methods were beyond the scope of this study. Nevertheless, it should be noted that the latter may elicit a stronger mucosal immune response. At present, the preclinical evaluation of norovirus vaccines has mainly focused on the histo blood group antigen (HBGA) blockade, but this approach does not fully reflect the immunogenicity of vaccines in vivo. In recent years, select human norovirus genotypes have been successfully cultured in human intestinal enteroids (HIEs). This non-transformed human intestinal organoid model, derived from surgical resections of the small intestine, mimics the human intestinal epithelium with multiple cell types and permits limited human norovirus replication, and provides a valuable platform for evaluating serum neutralizing antibodies (nAbs), which can effectively reflect the immunogenicity of vaccines in vivo [50]. Therefore, it is recommended that we evaluate human intestinal enteroids first, while using the NHP model only for vaccines that show the desired immunogenicity. This phased evaluation strategy can screen potential vaccine candidates at an early stage, while reducing unnecessary resource expenditure as well as alleviating ethical concerns related to NHP models.

The vaccinia virus has a long and storied history as the first preventive inoculation against the smallpox virus, against which it can provide lifelong protection with only one dose. However, vaccinia vector vaccines generally require a two-dose immunization. A study of vaccinia vector Ebola vaccines found that the first injection of the vaccine elicited only limited titers of antibodies, which were significantly enhanced after the booster dose. This result is consistent with our study, as we also found that the booster dose noticeably enhanced the VG9-NOR-induced immune responses, especially the antibodies blocking HBGA. GII.4-IgG- and -IgA-binding Abs, as well as GII.17-IgA-binding Ab increased after booster injection, but there was no significant change in GII.17-IgG-binding Ab between the first and second immunizations. We observed that intranasal immunization resulted in higher IgG -and IgA-binding Abs titers, as well as fecal IgA Abs positivity rates, compared with the intramuscular route. Moreover, increasing the vaccine dose significant improved both the humoral and cellular immune response, which is feasible due to the low toxicity of VG9.

## Data Availability

The data are contained within the article and supplementary materials.

## Supplementary Materials

The following supporting information can be downloaded at: https://www.mdpi.com/article/10.3390/v17020237/s1, Figure S1: Safety evaluation of VG9 and VG9-NOR; Figure S2: Expression validation; Figure S3: Passaging stability.
