# Supplementary material for: Vaccinia Virus Vector Bivalent Norovirus Vaccine
Source: Viruses. 2025 Feb 9;17(2):237. doi: 10.3390/v17020237 (PMC11861675; doi:10.3390/v17020237)
Supplement: Supplementary file 1 [file viruses-17-00237-s001.zip › viruses-3418520-supplementary.pdf]

Supplementary data
Vaccinia vaccine vector bivalent norovirus vaccine

Safety evaluation of VG9 and VG9-NOR. Organ pathological sections 2000um.

	liver	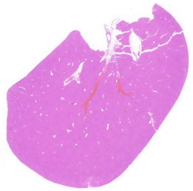		liver	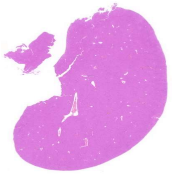		liver	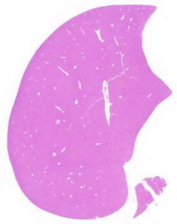
VG9	heart	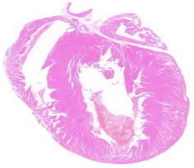	VG9-NOR（IM）	heart	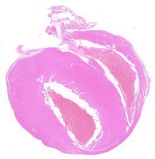	VG9-NOR (IN)	heart	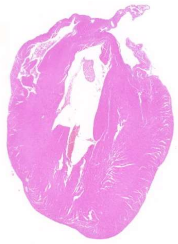
	brain	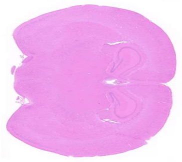		brain	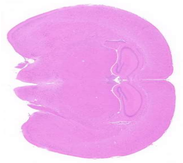		brain	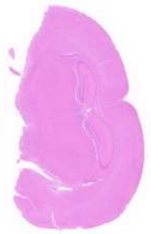

	kidney	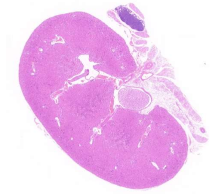		kidney	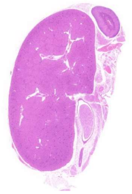		kidney	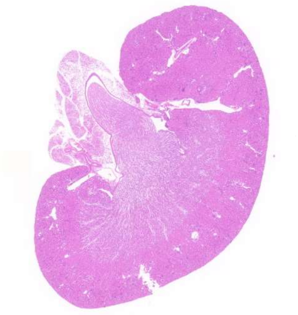

Expression Validation

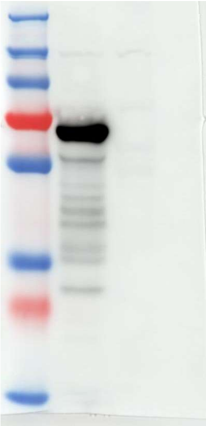

anti GII.4 VP1

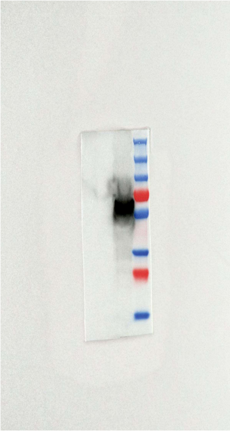

anti GII.17 VP1

Passaging Stability

	GADPH	VP1	VG9-E3
GII.4	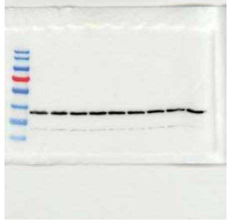	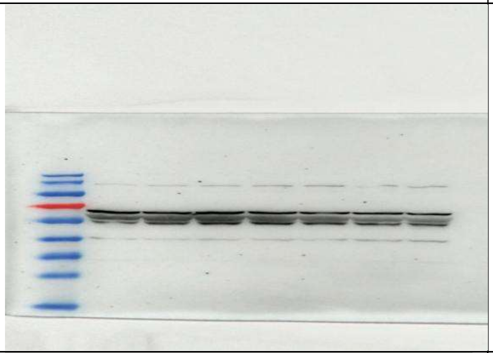	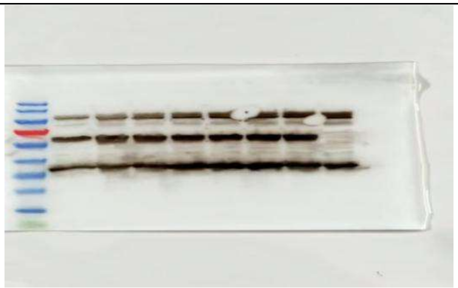
G11.17	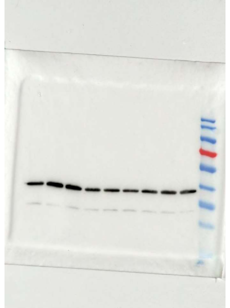	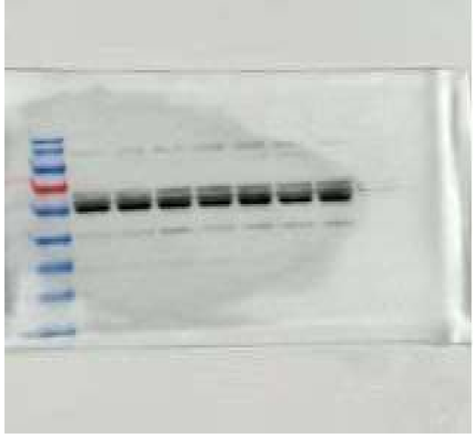	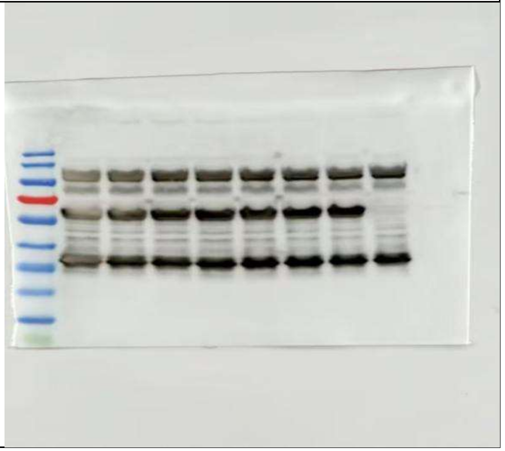
